# Silicon Fertilization: A Step towards Cadmium-Free Fragrant Rice

**DOI:** 10.3390/plants10112440

**Published:** 2021-11-12

**Authors:** Qamar uz Zaman, Muhammad Rashid, Rab Nawaz, Afzal Hussain, Kamran Ashraf, Maria Latif, Abdihakim Osman Heile, Faisal Mehmood, Sughra Salahuddin, Yinglong Chen

**Affiliations:** 1 Department of Environmental Sciences, The University of Lahore, Lahore 54590, Pakistan; rab.nawaz@envs.uol.edu.pk (R.N.); afzal.hussain@envs.uol.edu.pk (A.H.); maria.latif@envs.uol.edu.pk (M.L.); abdihakimosman4@gmail.com (A.O.H.); salahsughra@gmail.com (S.S.); 2 Nuclear Institute for Agriculture & Biology, Faisalabad 38000, Pakistan; drmrashidhust@gmail.com; 3 Department of Food Science and Nutrition, Government College University Faisalabad Sahiwal Campus, Sahiwal 57000, Pakistan; kamran2417@gmail.com; 4 Department of Chemistry, The University of Lahore, Lahore 54590, Pakistan; faisalmehmood186@gmail.com; 5 The UWA Institute of Agriculture, and School of Agriculture and Environment, The University of Western Australia, Perth, WA 6009, Australia; 6 Institute of Soil and Water Conservation, Chinese Academy of Sciences, and Northwest Agriculture & Forestry University, Yangling, Xianyang 712100, China

**Keywords:** cadmium accumulation, Cd stress, grain yield, physiological traits, rice, silicon fertilizer

## Abstract

Soil contamination with toxic cadmium (Cd) is becoming a serious global problem and poses a key hazard to environments and the health of human beings worldwide. The present study investigated the effects of foliar applications of three forms of silicate chemicals (calcium silicate, sodium silicate, and potassium silicate) at four rates (0.25%, 0.5%, 0.75%, and 1.0%) at tillering stage on rice growth and the accumulation of Cd under Cd stress (30 mg kg^−1^). The results showed that Cd stress reduced the yield-related traits and enlarged Cd contents in different rice organs. The leaf gas exchange attributes and yield traits were enhanced, and the Cd accumulation and bioaccumulation factor in rice organs were reduced, especially in grains, through silicon application. In shoots, roots, and grains, foliar spray of Si reduced Cd contents by 40.3%, 50.7%, and 47.9%, respectively. The effectiveness of silicate compounds in reducing Cd toxicity varied with the kind of chemicals and doses of foliar applications. Foliar application of potassium silicate, at a rate of 0.5%, at tillering stage, showed the best effectiveness in improving grain yield, while mitigating Cd accumulation in rice grains. The outcome of this study provides a promising practicable approach in alleviating Cd toxicity in rice and preventing the entrance of Cd into the food chain.

## 1. Introduction

In terrestrial ecological systems, soil is a critical supporting medium, as well as a key source of essential minerals and nutrients for plants growth; additionally, it is a part of material and energy diffusion [1]. Environmental contamination, due to heavy metals pollution, has become an alarming issue in many countries. Cadmium (Cd), lead (Pb), chromium (Cr), and nickel (Ni) are normally the presented toxic heavy metals in agricultural lands [2,3]. Soil contamination, through heavy metals, is a main environmental issue and contamination of agricultural fields with Cd is of greater concern [4,5,6]. Cd is a highly toxic metal for plants, animals, and human beings [7,8]. Cadmium is very mobile, which is why it can enter into our food chain and pose adverse health-related effects [9]. For many countries, these agricultural soils have been contaminated with Cd from irrigation, due to waste, effluent from different kinds of chemical and fertilizers industries, mining, and landfills [10,11]. Cadmium toxicity resulted in harmful effects in plants, such as growth inhibition, deficiency of photosynthesis pigments, leaf chlorosis, carbohydrate alteration, oxidative stress, imbalance of homeostasis, and lower crops yields [12]. Thus, Cd stress adversely influences plant growth, grain yield, and food quality [13,14]. On the other hand, when raw-effluent, polluted with metals, is applied to food crops, these toxic metals enter into plants’ edible parts and cause adverse effects to those who consume such contaminated crops [15,16].

Among cereal crops, Rice is a major crop, and about half world population depends on rice as its basic food [17,18,19,20,21]. Cd enters plants through the root cortical tissues and travels into the xylem via a symplastic and/or apoplastic pathway before entering the xylem part of the roots. Consequently, Cd is translocated towards the shoots and grains, causing toxicity to human beings who consume Cd-contaminated grains [22,23,24]. Therefore, there is an urgent need to take measures, so Cd could not be taken up by plants and to prevent its translocation towards grains.

Some cost-effective and environmentally friendly approaches, such as biochar, farmyard manure, organic acids, nano-materials, etc., have been attempted metal contaminated soil reclamation, in order to diminish Cd accumulation in plant tissues, in particular grains, under Cd-contaminated land [25,26,27,28,29]. The use of silicon (Si) shows promise in elevating plant responses to abiotic stress, including metal toxicity [15,30]. Silicon is abundant in our soils, but it is not bioavailable, so the use of bioavailable forms of Si is essential to cope with plant stress (when grown in Cd-contaminated soil) [31]. Previous findings have been shown that Si-mediated metal toxicity alleviation can mainly attribute to reduced intake and transportation of Cd among plant organs in cotton [32], Chinese cabbage [33], wheat [12], peanut [34], and rice [35]. Si decreases the toxicity and Cd accumulation in rice, typically, Si-accumulating crop variety. Calcium silicate decreased the Cd concentration in straw and grains of rice [36], such as 24% deduction in rice shoots [37].

Previous studies involving Si are often limited to the use of one type of Si salts and a single dose. Whether other forms of Si salts are advanced and whether combined use of multiple Si salts, under appropriate levels, are more effective for alleviating Cd stress in rice remains unknown. Hence, the objectives of the current study were: (1) to evaluate the impact of different silicate chemicals (calcium silicate, sodium silicate, and potassium silicate), under varying dose levels, on the growth and productivity of rice in Cd-contaminated soils; and (2) to assess the efficiency of these chemicals for the alleviation of Cd stress and reduction of Cd accumulation in rice grains.

## 2. Results

### 2.1. Growth Attributes

Analysis of variance showed that various types of silicate chemicals (C), different levels (T), and the interactive effect of C × T meaningfully (*p* ≤ 0.01) affected the root, shoot length, and rice plants height grown in Cd-spiked soil (Table 1). For silicate chemicals, maximum root length (6.8 cm), shoot length (17.6 cm), and plant height (75.8 cm) were observed in the treatment of potassium silicate, followed by sodium silicate and then calcium silicate. Different foliar application treatments also showed variation in all growth attributes. Maximum increases in the root length (76.9%), shoot length (78.9%), and plant height (53.0%) were observed where foliar application of 0.50% of potassium silicate solution was applied, as compared to the control (Table 1).

### 2.2. Photosynthetic Attributes

**Figure 1:**
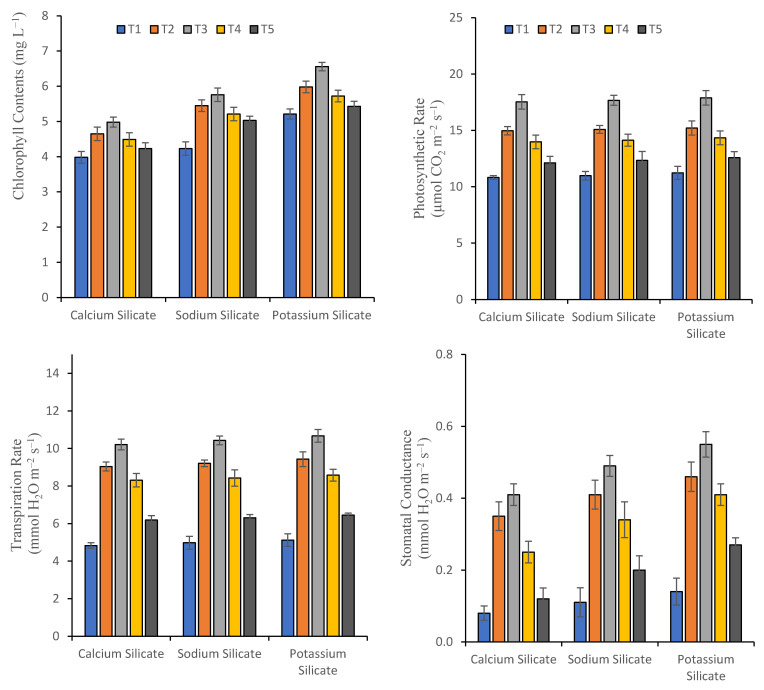
Effect of silicate chemicals, with various doses of foliar applications, on the photosynthetic attributes of rice grown in Cd-contaminated soil.

The exogeneous application of Si significantly improved the photosynthetic attributes (i.e., transpiration rate, photosynthetic rate, stomata conductance, and chlorophyll contents) in the plants of rice grownup within Cd-spiked soil (Figure 1). Maximum increases in chlorophyll contents (15.7%), photosynthetic rate (0.79%), transpiration rate (2.15), and stomatal conductance (19.51%) were observed when sodium silicate was applied at the rate of 0.50%, as compared to calcium silicate. However, potassium silicate, when applied at same concentration, improved the chlorophyll contents (31.7%), photosynthetic rate (2.05%), transpiration rate (4.50%), and stomatal conductance (34.1%), as compared to calcium silicate. However, application of potassium silicate, at the rate of 0.50%, significantly improved the photosynthetic attributes, as compared to the control.

### 2.3. Yield Attributes

Applications of Si in rice showed a significant impact (*p* ≤ 0.01) on yield attributes of rice, as compared to non-Si treatment (Table 2). All the interactive effects were non-significant results for all the yield attributes, except for panicle length, 100-kernels weight, and grain yield. Maximum panicle length (17.5 cm), number of kernels per panicle (49.3), 100-kernels weight (16.8 g), yield of straw (20.6 g plant^−1^), grain yield (10.55 g plant^−1^), biological yield (31.1 g plant^−1^), and harvest index (32.4%) were observed where potassium silicate was applied, as compared to sodium and calcium silicate, while the minimum of all yield attributes was observed where calcium silicate was applied in Cd-spiked soil. For foliar application rates, the maximum increases in number of tillers per hill (80.6%), panicle length (59.4%), number of kernels per panicle (53.3%), 100-kernels per panicle (59.1%), straw yield (88.2%), and grain yield (95.8%) were noticed where 0.50% foliar application was applied in the soil, as compared to the control.

### 2.4. Cd and Si Accumulations

In roots, shoots, and grains of rice, significant differences in Cd and Si contents were observed (Table 3). The interactive effect of C × T was significant for Cd content in roots and shoots, respectively. For silicate chemicals, the maximum Cd concentration in roots (7.77 mg kg^−1^), shoots (6.85 mg kg^−1^), and grains (1.80 mg kg^−1^) was observed in C_1_ (calcium silicate) and minimum Cd concentration in roots (7.16 mg kg^−1^), shoots (5.14 mg kg^−1^), and grains (1.54 mg kg^−1^) was noticed in C_3_ (potassium silicate). Maximum decrease of Cd contents in roots (50.7%), shoots (40.3%), and grains (47.9%) were noticed, where foliar application of Si was performed at the rate of 1.00% of potassium silicate. Similarly, maximum Si contents were observed, where Si foliar application was performed at the rate of 1.00%, using the same chemical. The decreasing order for the reducing Cd contents and improving Si contents for the silicate compounds was potassium silicate > sodium silicate > calcium silicate (Table 3). In the treatment where Si application was performed (at the rate of 0.50%), under the Cd-spiked soil, the lowermost concentrations of Cd in rice grains, shoots, and roots were found (Table 3). The results showed that exogeneous applied Si diminished the concentration of Cd in the roots, shoots, and grains of rice.

### 2.5. Health Risk Index

The health risk index (HRI) values of Cd, by the food chain for adults, were decreased with Si application, as compared to the control (Table 4). With respect to adults, the HRI values ranged from 0.20 to 0.44, as maximum value for the control and the smallest value for Si application, in the form of potassium silicate, at a spray rate of 1.00%.

## 3. Discussion

Globally, agricultural soils contaminated by Cd, lead to a greater reduction in growth and productivity of plant [38]. In plants, Cd toxicity varies with the growth conditions, experimental settings, and depends on the availability of Cd, exposure time, and of the plant growth stages [39]. The present study revealed that the growth attributes of rice plants were considerably suppressed by the cadmium stress. However, the exogenous Si (potassium silicate) application, at the rate of 0.50% at tillering stage, improved the growth attributes under Cd stress. However, application rates above 0.50% cause toxic effects on leaves. These same observations were recorded in previous studies [40]. Under Cd stress, the application of Si may improve the plant growth in various ways, such as through increasing nutrient levels, chlorophyll contents, root exudates of organic acids, root volume, and overall root growth [15]. The simulated root growth by potassium silicate could be due to the reason that potassium is more effective for the activation of enzymes, which enhanced plant growth and development, compared with other forms of silicate salts. In addition, Si would reduce the Cd toxic impacts, as well as additional metals, through improving the defense mechanisms of the plant. It is enhanced through decreasing the production of reactive oxygen species (ROS) and the antioxidant defense system improvement [41]. However, in rice, the ultrastructure changes of chloroplast, posed with Cd stress, were positively affected by Si [40]. To mitigate Cd stress in plants, Si treatment played vital role by improving the growth of rice.

Among the foremost physiological processes that are extremely sensitive to metal stress, photosynthesis is one of the major one [7]. In the current investigation, under Cd stress, total chlorophyll contents and photosynthesis attributes were reduced in the plants grown in non-Cd-contaminated soil (Figure 1). Findings of the earlier research also documented that photosynthesis inhibited by Cd in plants [40,42]. In the presence of Cd, the reduction in photosynthetic attributes might be by the reason of the Cd negative consequences on photosynthetic machinery [7,40]. With the exogeneous application of Si the negative effect on photosynthesis is mitigated. In plants, improvements in concentrations of chlorophyll and light use efficiency might be the reason for the increase in photosynthetic performance in Si-mediated plants [40]. It has been documented that, due to the formation of double layer in the plant leaves, the transpirations reduced, and the water use efficiency boosted [43]. Therefore, by the Si application, rice Cd toxicity is decreased, thus improving the photosynthetic capacity of plants.

Yield of rice plants involve a long-term, continuous method. Through this method, the plants in nature rely mostly on their own ability to resist the toxic effect from Cd [44]. Application of Si improved the yield attributes of rice by reducing the Cd toxicity (Table 2). These same observations were recorded in previous studies [45]. The improvement in the yield attributes might be due to enhancement in the essential nutrient’s elements in root volume, chlorophyll contents, and discharge of organic acids and histological features improved by the silicon application under metal stress [46]. Cd toxicity and toxic consequences on rice can be alleviated by Si binding protein and mainly reduced antioxidant activity [37]. The toxic effect of Cd (by enhancing the antioxidant defense system) and plant protection (by decreasing Si and increasing ROS production) ultimately results in the best yield performance under the Cd stress [47]. The yield improvement in rice plants could be attributed to the potential of Si to increase growth and concentration of photosynthetic pigments and photosynthesis by induced Si [48]. The stunted growth of rice plants and increased the number of empty grains in kernels significantly deceased grain yield and might be the reason of Cd stress and silicon deficiency [49].

Greater metals and metalloids accumulation in roots, rather than in other plant organs in the crops, are observed in previous studies [50,51,52,53]. In the roots by many factors, such as compartmentalization of vacuolar sequestration, apoplastic barriers, and chelation restricts the translocation of Cd by the higher amount in the shoot and grain portion of rice plant [54]. The translocation of Cd from roots to shoots can be reduced by the increase in production of thiol in roots [46,55]. The process of Cd-alleviation by Si has been studied, and it appears that a large deposition of Si near endodermis may contribute to increased Cd-retention in cell walls [56]. According to a recent study, the complexation and co-deposition of Si-hemicellulose with Cd may occur, resulting in Cd uptake inhibition in rice cells [57]. Furthermore, the role of Si in reducing the deleterious effects of Cd-stress on plants involves changes in the expression of multiple genes, as well as the metabolism [10].

**Figure 2:**
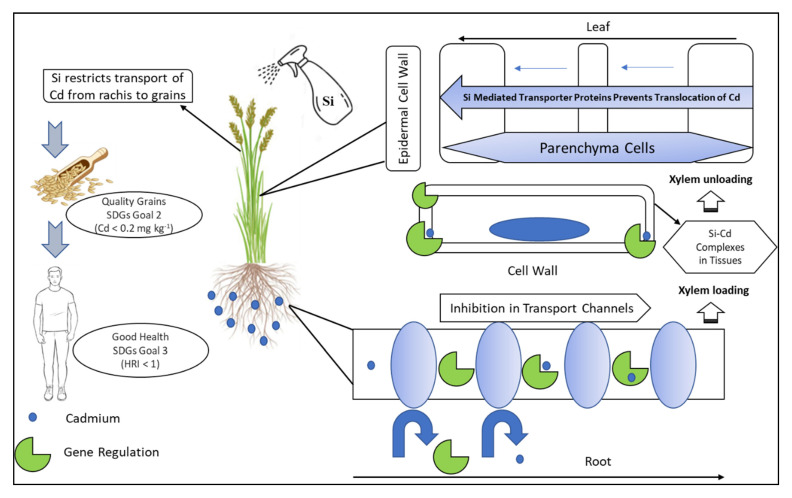
A schematic diagram of uptake, transport, and accumulation of Si in rice plant. Soil applied Si is absorbed by the roots to the root exodermis by the influx transporter genes and afterward released to the apoplast by the efflux transporter genes. However, exogeneous application of Si is translocated into the xylem and transported via the transpiration pull to the shoots. In the leaves, Si is localized in the xylem parenchyma cells of leaf and unloaded by transporter genes. That ultimately results in restriction of Cd entry from rachis to grains producing quality rice for good health.

Several potential mechanisms have been proposed for the function of Si in the alleviation of Cd stress in plants: (i) Si-induced decrease in Cd accumulation, including decreased apoplastic transport of Cd, due to Si deposition in the endodermis and epidermis cell wall [37], (ii) the formation of a [Si–hemicellulose matrix] Cd complex and succeeding co-precipitation [57], (iii) down-regulation of genes involved in Cd accumulation [57], and (iv) reduce supply of Cd soil due to pH increase after application of silicate compounds for achieving the good quality grains for achieving the best health [58] as depicted in (Figure 2).

The health risk of any toxic chemical can be assessed by quantifying the possible quantity and route of entry to the target organisms. There are several routes through which Cd can be enter to humans but the major pathway of Cd entry is the food chain [7]. With respect to the critical Cd limits (0.2 mg kg^−1^) in cereal grains, the exogeneous application of Si reduced the concentration of Cd in the grains below the threshold level under the conditions of experiment (Table 4). These findings verified Si application may result in the reduction of the Cd concentration in grains. Si reduced the HRI for Cd, as shown in the results (Table 4). These outcomings showed that supply of Si not only enhances rice grains quantity but also the health rick lessened by it by the use of food with contamination of Cd. Our findings showed that HRI values are lower than one even for control, but that they may exceed the stated limit if Cd-contaminated rice grains are consumed for a longer period of time. As a result, there is a need to address metal-contaminated areas, and the exogeneous application of Si could be a viable solution in this regard.

## 4. Materials and Methods

### 4.1. Experimental Design and Treatments

The pot experiment was carried out in a naturally-lit glasshouse at the Department of Environmental Sciences, The University of Lahore, Pakistan. A completely randomized design (CRD) was used in this study comprised of two factors: silicon compounds (calcium silicate [Ca_2_O_4_Si], sodium silicate [Na_2_SiO_3_], and potassium silicate [K_2_O_3_Si]) and application doses (0, 0.25%, 0.50%, 0.75% and 1.0%), with three replications.

### 4.2. Plant Materials, Experiment Setup, and Maintenance

A local wide-planted rice cultivar Super Basmati was used as test cultivars. Sodium hypochlorite (2.6% active chloride) was used to surface sterilize rice seeds for 2 min and then using deionized water washed carefully for three times. Seeds were sown in farm soil without Cd amendment in a nursery for 25 days before transplanted in to pots (15 cm diameter and 20 cm depth) filled with 10 kg of air-dried farm soil with CdCl_2_ amendment (at the rate of 30 mg kg^−1^). Initially, ten uniform seedlings were transplanted into each pot and thinned to six per pot after 7 days. Fertilizers containing N: P: K at suggested doses rate (140:90:60 kg ha^−1^) were applied before to transplantation by mixing to the soil, i.e., 1.61 g pot^−1^ diammonium phosphate (DAP), 0.63 g pot^−1^ sulfate of potash (SOP), and 0.20 g pot^−1^ urea (first N split), though residual N application, was performed at tillering and booting initiation (0.2 g pot^−1^), respectively. Pots were irrigated with tap water throughout the whole growth period, and a 2 cm layer of water was sustained on soil surface, excluding in latter growth periods.

For silicon treatments, solution of each silicon compound was prepared based on the required amount foreach treatment by adding the chemical in a flask containing small amount of distilled water dispersed for about 30 min using ultra-sonication. The final volumes were made of each treatment to get the desired concentrations of each treatment. After transplanting the first exogeneous application of silicate chemicals was performed 20 days after transplanting and remaining three foliar applications were performed at 1-week intervals using a small hand sprayer. For each foliar spray to subsequent treatments freshly prepared 2.0 L of Si solution was used. Distilled water was used for the foliar spray of control plants. Rice plants were assessed at the vegetative (55 days after transplanting, DAT) and at physiological maturity stages (four months after transplanting).

### 4.3. Measurements and Data Collection

On the 55 DAT, between 9:00 a.m. to 12:00 noon, the fully expanded upper most leaves were used for stomatal conductance (gs), photosynthetic rate (A), and transpiration rate (E), using a portable photosynthesis system infrared gas analyzer (IRGA) (Analytical Development Company, Hoddesdon, England, UK). For chlorophyll contents, the protocols of Nagata and Yamashita, [59] were used. Fresh leaves were cut into segments of 0.5 cm and at −10 °C extracted overnight with 80% acetone. The extract was centrifuged for 5 min at 14,000× *g* rpm and supernatant absorbance was read at 645 and 663 nm using a spectrophotometer (Halo DB-20/Db-20S, Dynamica Company, London, UK). On the 55 DAT, three plants from each treatment (one plant per pot) were randomly selected and pulled out for measuring root and shoot length, with the help of measuring scale.

At the physiological maturity, three plants were randomly selected and tagged from every pot for plant height measurements with a measuring scale. After harvesting of all plants in each pot, the tagged plants were used for counting tiller number per pot. Randomly selected five panicles of tillers from each pot were used counting of branches per panicle. Kernels per panicle were counted and 100-grain weight of kernels was obtained in grams by using an automatic electric balance after air drying. After threshing, the clean rough rice grains were air-dried and weighed with 14% moisture content. Biological yield per pot was determined by adding the grain yield and straw yield per pot. Straws were sun-dried for one week and straw dry weight per pot was determined. Harvest index was determined by the proportion of grain yield to biological yield and stated on a percentage basis.

### 4.4. Quantification of Cd and Si Contents in Plant Tissues

For the measurement of Cd concentration in dried root, shoot, and grain tissues at physiological maturity, a mixture of di-acid method was practiced by the subsequent procedures of Jones and Case, [60]. A Perkin-Elmer for atomic absorption spectrophotometer (novAA ^®^ 350- Analytik Jena, Jena, Germany) was used to evaluate the concentration of Cd [61]. For the estimation of Si contents, the rice plant parts sample (<0.250 mm) was digested in a mixture of 62% (*w/w*) HNO_3_ (3 mL), 30% (*w/w*) hydrogen peroxide (3 mL), and 46% (*w/w*) HF (2 mL). After that, 4% (*w/v*) boric acid was used to dilute the digested sample to 100 mL. Colorimetric molybdenum blue method at 600 nm was used to determine the Si concentration in the digest solution [62].

### 4.5. Health Risk Index

The human health risk index (HRI) of Cd was calculated by measuring the daily intake of metal (DIM) and dividing it with oral reference doses (RFD) of Cd.

HRI = DIM/RFD

The RFD value for Cd is reported as 0.001 mg kg^−1^ body weight/day [63]. Daily intake of Cd was estimated by multiplying the concentration of Cd in grains with daily food intake and conversion factor and dividing by average body weight of person. By taking the average body weight in Pakistan (65 kg), the average rice consumption per capita 0.15 kg day^−1^ [64].

*DI*M = Cd in grain × Daily Food intake of rice × conversion factor/average body weight.

A HRI index value of more than one is considered unsafe for human health [65].

### 4.6. Statistical Analysis

Statistical analysis of data was performed by using Statistix 8.1 (Tallahassee, FL, USA, 1985–2003). To compare the treatments means, the highest significance difference (HSD) test, at a probability level of 5%, was applied. Graphs were made in Microsoft Excel software using means ± S.E.

## 5. Conclusions

The current study revealed that the exogenous application of Si enhanced the rice growth and biomasses, chlorophyll contents, photosynthesis, and antioxidants enzyme activities by lowering the oxidative stress and Cd uptake of rice plants. Each single type of Si salt restricted Cd uptake, with the potassium silicate the most efficient Si salt, compared to calcium silicate and sodium silicate. Thus, we recommend that the optimum dose of potassium silicate has the potential to minimize Cd uptake in rice. Moreover, reduction in Cd accumulation in the grains ensures rice quality, which would further reduce the HRI. These are amongst the main sustainable development goals (SDGs) for good health and zero hunger. However, further studies, including field investigations in various environments, are needed prior to the large-scale application of potassium silicate.

## Figures and Tables

**Table 1:** Effect of silicate chemicals, with various doses of foliar applications, on the growth attributes of rice grown in Cd-contaminated soil.

Treatments	Root Length (cm)	Shoot Length (cm)	Plant Height (cm)
Silicate Chemicals (C)			
C_1_ = Calcium Silicate	5.7 C	12.5 C	66.9 C
C_2_ = Sodium Silicate	6.5 B	14.9 B	70.8 B
C_3_ = Potassium Silicate	6.8 A	17.6 A	75.8 A
HSD (C) (*p* ≤ 0.01)	0.31	0.30	0.29
Foliar Application Treatments (T)			
T_1_ = 0 (Control)	4.7 E	11.4 E	55.3 E
T_2_ = 0.25%	7.4 B	18.2 B	79.5 B
T_3_ = 0.50%	8.4 A	20.4 A	84.6 A
T_4_ = 0.75%	6.8 C	14.6 C	72.1 C
T_5_ = 1.00%	5.9 D	12.8 D	64.6 D
HSD (T) (*p* ≤ 0.01)	0.47	0.46	0.44
Significance Level (C)	40.2 **	860 **	2849 **
Significance Level (T)	280 **	1564 **	11,697 **
Significance Level (C × T)	4.15 **	98.5 **	219 **

Within each column, mean data followed by the same letters are not statistically different (*p* ≤ 0.01 HSD test). **, *p* < 0.01.

**Table 2:** Effect of silicate chemicals, with various doses of foliar applications, on the yield attributes of rice grown in Cd-contaminated soil.

Treatments	Number of Tillers per Hill	Panicle Length (cm)	Number of Kernels per Panicles	100-Kernels Weight (g)	Straw Yield (g pant^−1^)	Grain Yield (g plant^−1^)	Biological Yield (g plant^−1^)	Harvest Index (%)
Silicate Chemicals (C)								
C_1_ = Calcium Silicate	6.20	14.5 C	43.7 B	14.5 C	16.5 B	7.22 C	23.7 B	29.7
C_2_ = Sodium Silicate	8.13	16.3 B	45.7 B	15.6 B	18.0 AB	8.95 B	27.0 AB	31.9
C_3_ = Potassium Silicate	8.93	17.5 A	49.3A	16.8 A	20.6 A	10.6 A	31.1 A	32.4
HSD (C) (*p* ≤ 0.01)	2.96	0.31	3.13	0.34	3.78	1.15	4.18	4.25
Foliar Application Treatments (T)								
T_1_ = 0 (Control)	5.66 B	12.8 E	37.1 D	12.3 E	13.1 C	6.71 E	16.8 C	22.1 C
T_2_ = 0.25%	9.44 AB	18.2 B	51.4 B	17.6 B	21.7 AB	12.2 B	33.9 A	36.3 A
T_3_ = 0.50%	10.2 A	20.4 A	56.9 A	19.6 A	24.7 A	13.1 A	38.8 A	36.7 A
T_4_ = 0.75%	7.22 AB	15.4 C	45.2 C	15.3 C	17.8 BC	9.70 C	26.5 B	33.2 AB
T_5_ = 1.00%	6.22 AB	13.6 D	40.3 D	13.5 D	14.6 C	7.79 D	20.4 BC	28.3 BC
HSD (T) (*p* ≤ 0.01)	4.50	0.47	4.75	0.52	5.74	0.74	6.35	6.45
Significance Level (C)	2.73 ^NS^	284 **	9.97 **	2.73 **	3.63 *	25.4 **	9.62 **	1.39 ^NS^
Significance Level (T)	3.30 *	736 **	48.3 **	3.30 **	11.8 **	103 **	34.8 **	15.2 **
Significance Level (C × T)	1.03 ^NS^	36.7 **	1.66 ^NS^	1.03 **	0.33 ^NS^	2.26 *	0.79 ^NS^	0.59 ^NS^

Within each column, mean data followed by the same letters are not statistically different (*p* ≤ 0.01 HSD test). *, *p* < 0.05; **, *p* < 0.01; NS, not significant.

**Table 3:** Effect of silicate chemicals, with various doses of foliar applications, on the Cd and Si contents (mg kg^−1^) in rice organs grown in Cd-contaminated soil.

Treatments	Cd in Root	Cd in Shoot	Cd in Grain	Si in Root	Si in Shoot	Si in Grain
Silicate Chemicals (C)						
C_1_ = Calcium Silicate	7.77 A	6.85 A	1.80 A	2.17 C	1.76 C	0.36 B
C_2_ = Sodium Silicate	7.38 AB	5.77 B	1.68 B	2.27 B	1.81 B	0.44 A
C_3_ = Potassium Silicate	7.16 B	5.14 C	1.54 C	2.37 A	1.98 A	0.51 A
HSD (C) (*p* ≤ 0.01)	0.57	0.34	0.08	0.05	0.05	0.07
Foliar Application Treatments (T)						
T_1_ = 0 (Control)	10.1 A	7.76 A	2.17 A	1.56 E	1.27 E	0.18 E
T_2_ = 0.25%	7.97 B	6.59 B	1.97 B	1.86 D	1.46 D	0.30 D
T_3_ = 0.50%	7.23 BC	5.88 C	1.71 C	2.14 C	1.87 C	0.41 C
T_4_ = 0.75%	6.82 C	4.74 D	1.41 D	2.69 B	2.13 B	0.58 B
T_5_ = 1.00%	5.00 D	4.63 D	1.13 E	3.10 A	2.52 A	0.71 A
HSD (T) (*p* ≤ 0.01)	0.87	0.52	0.12	0.08	0.08	0.10
Significance Level (C)	3.46 *	77.7 **	28.5 **	51.6 **	39.4 **	12.3 **
Significance Level (T)	76.1 **	107 **	176**	599 **	949 **	63.7 **
Significance Level (C × T)	15.4 **	14.2 **	1.97 ^NS^	3.33 **	1.16 ^NS^	0.78 ^NS^

Within each column, mean data, followed by the same letters, are not statistically different (*p* ≤ 0.01 HSD test). *, *p* < 0.05; **, *p* < 0.01; NS, not significant.

**Table 4:** Effect of silicate chemicals, with various doses of foliar applications, on the HRI of rice grown in Cd-contaminated soil.

Treatment	Calcium Silicate	Sodium Silicate	Potassium Silicate
T_1_	0.44	0.44	0.43
T_2_	0.41	0.40	0.38
T_3_	0.37	0.36	0.31
T_4_	0.34	0.27	0.25
T_5_	0.26	0.23	0.20

T_1_ = control; T_2_ = foliar application of Si @ 0.25%; T_3_ = foliar application of Si @ 0.50%; T_4_ = foliar application of Si @ 0.75%; T_5_ = foliar application of Si @ 1.00%.

## Data Availability

Data are contained within the article.

## References

[1] Wang Y., Luo W., Zeng G., Peng H., Cheng A., Zhang L., Cai X., Chen J., Lyu Y., Yang H. (2020). Characteristics of carbon, water, and energy fluxes on abandoned farmland revealed by critical zone observation in the karst region of southwest China. Agric. Ecosyst. Environ..

[2] Hussain A., Ali S., Rizwan M., Rehman M.Z.U., Hameed A., Hafeez F., Alamri S., Alyemeni M.N., Wijaya L. (2018). Role of Zinc–Lysine on Growth and Chromium Uptake in Rice Plants under Cr Stress. J. Plant Growth Regul..

[3] Shah V., Daverey A. (2021). Effects of sophorolipids augmentation on the plant growth and phytoremediation of heavy metal con-taminated soil. J. Clean. Prod..

[4] Czyżewski B., Trojanek R., Dzikuć M., Czyżewski A. (2020). Cost-effectiveness of the common agricultural policy and environ-mental policy in country districts: Spatial spillovers of pollution, bio-uniformity and green schemes in Poland. Sci. Total Environ..

[5] Wang J., Wang L., Wang Y., Tsang D.C., Yang X., Beiyuan J., Yin M., Xiao T., Jiang Y., Lin W. (2020). Emerging risks of toxic metal(loid)s in soil-vegetables influenced by steel-making activities and isotopic source apportionment. Environ. Int..

[6] Hasan A.B., Reza A.S., Kabir S., Siddique M.A.B., Ahsan M.A., Akbor M.A. (2020). Accumulation and distribution of heavy metals in soil and food crops around the ship breaking area in southern Bangladesh and associated health risk assessment. SN Appl. Sci..

[7] Rizwan M., Ali S., Hussain A., Ali Q., Shakoor M.B., Rehman M.Z.U., Farid M., Asma M. (2017). Effect of zinc-lysine on growth, yield and cadmium uptake in wheat (*Triticum aestivum* L.) and health risk assessment. Chemosphere.

[8] Ehsan N., Qamar K.M., Zaman Q., Ahmed W., Aslam A., Mehmood F. (2020). Sustainable remediation solution for heavy metal contaminated soils of Pakistan: A review. Pure Appl. Biol..

[9] Rigby H., Smith S.R. (2020). The significance of cadmium entering the human food chain via livestock ingestion from the agricultural use of biosolids, with special reference to the UK. Environ. Int..

[10] Kim Y.-H., Khan A.L., Kim D.-H., Lee S.-Y., Kim K.-M., Waqas M., Jung H.-Y., Shin J.-H., Kim J.-G., Lee I.-J. (2014). Silicon mitigates heavy metal stress by regulating P-type heavy metal ATPases, *Oryza sativalow* silicon genes, and endogenous phytohormones. BMC Plant Biol..

[11] Sanaei F., Amin M.M., Alavijeh Z.P., Esfahani R.A., Sadeghi M., Bandarrig N.S., Fatehizadeh A., Taheri E., Rezakazemi M. (2020). Health risk assessment of potentially toxic elements intake via food crops consumption: Monte Carlo simulation-based probabilistic and heavy metal pollution index. Environ. Sci. Pollut. Res..

[12] Rizwan M., Meunier J.-D., Davidian J., Pokrovsky O.S., Bovet N., Keller C. (2016). Silicon alleviates Cd stress of wheat seedlings (*Triticum turgidum* L. cv. Claudio) grown in hydroponics. Environ. Sci. Pollut. Res..

[13] Jallad K.N. (2015). Heavy metal exposure from ingesting rice and its related potential hazardous health risks to humans. Environ. Sci. Pollut. Res..

[14] Ke S., Cheng X.-Y., Zhang N., Hu H.-G., Yan Q., Hou L.-L., Sun X., Chen Z.-N. (2015). Cadmium contamination of rice from various polluted areas of China and its potential risks to human health. Environ. Monit. Assess..

[15] Hussain A., Rizwan M., Ali Q., Ali S. (2019). Seed priming with silicon nanoparticles improved the biomass and yield while reduced the oxidative stress and cadmium concentration in wheat grains. Environ. Sci. Pollut. Res..

[16] Hussain A., Rizwan M., Ali S., Rehman M.Z.U., Qayyum M.F., Nawaz R., Ahmad A., Asrar M., Ahmad S.R., Alsahli A.A. (2021). Combined use of different nanoparticles effectively decreased cadmium (Cd) concentration in grains of wheat grown in a field contaminated with Cd. Ecotoxicol. Environ. Saf..

[17] Kosolsaksakul P., Farmer J.G., Oliver I.W., Graham M.C. (2014). Geochemical associations and availability of cadmium (Cd) in a paddy field system, northwestern Thailand. Environ. Pollut..

[18] Meharg A.A., Norton G., Deacon C., Williams P., Adomako E., Price A., Zhu Y., Li G., Zhao F.-J., McGrath S. (2013). Variation in Rice Cadmium Related to Human Exposure. Environ. Sci. Technol..

[19] Zaman Q.U., Aslam Z., Yaseen M., Ihsan M.Z., Khaliq A., Fahad S., Bashir S., Ramzani P.M.A., Naeem M. (2018). Zinc biofor-tification in rice: Leveraging agriculture to moderate hidden hunger in developing countries. Arch. Agron. Soil Sci..

[20] Zaman Q., Aslam Z., Rashid M., Khaliq A., Yaseen M. (2018). Influence of zinc fertilization on morpho-physiological attributes, growth, productivity and hematic appraisal of paddy rice. J. Anim. Plant Sci..

[21] Zaman Q., Aslam Z., Rashid M., Aslam A., Ehsan N., Saqib Z.A., Yaseen M. (2020). Zinc nutrition application augments mor-pho-physiological attributes, productivity and grain zinc bioavailability of Paddy Rice. J. Appl. Bot. Food Qual..

[22] Aziz R., Rafiq M.T., Li T., Liu D., He Z., Stoffella P.J., Sun K., Xiaoe Y. (2015). Uptake of Cadmium by Rice Grown on Contaminated Soils and Its Bioavailability/Toxicity in Human Cell Lines (Caco-2/HL-7702). J. Agric. Food Chem..

[23] Lux A., Martinka M., Vaculík M., White P.J. (2010). Root responses to cadmium in the rhizosphere: A review. J. Exp. Bot..

[24] Naveed M., Saifullah, Riaz U., Murtaza G., Bibi S., Arooj A., Zaman Q.U. (2018). Strategic use of water: A step toward cadmium-free basmati rice (*Oryza sativa* L.). Paddy Water Environ..

[25] Ashraf S., Ali Q., Zahir Z.A., Ashraf S., Asghar H.N. (2019). Phytoremediation: Environmentally sustainable way for reclamation of heavy metal polluted soils. Ecotoxicol. Environ. Saf..

[26] Azeem M., Ali A., Jeyasundar P.G.S.A., Li Y., Abdelrahman H., Latif A., Li R., Basta N., Li G., Shaheen S.M. (2021). Bone-derived biochar improved soil quality and reduced Cd and Zn phytoavailability in a multi-metal contaminated mining soil. Environ. Pollut..

[27] Luo W., Ma J., Aman Khan M., Liao S., Ruan Z., Liu H., Zhong B., Zhu Y., Duan L., Fu L. (2021). Cadmium accumulation in rice and its bioavailability in paddy soil with application of silicon fertilizer under different water management regimes. Soil Use Manag..

[28] Radzali N., Kadir W., Shariff S.M., Nawahwi M.Z., Wakid S.A., Jaafar Z., Rahim M.I. (2015). Phytoremediation: Environmental-friendly clean up method. World.

[29] Tahir N., Ullah A., Tahir A., Rashid H.U., Rehman T.U., Danish S., Hussain B., Akca H. (2021). Strategies for reducing Cd concentration in paddy soil for rice safety. J. Clean. Prod..

[30] Ali S., Rizwan M., Hussain A., Zia Rehman M., Ali B., Yousaf B., Wijaya L., Alyemeni M.N., Ahmad P. (2019). Silicon nano-particles enhanced the growth and reduced the cadmium accumulation in grains of wheat (*Triticum aestivum* L.). Plant Physiol. Biochem..

[31] Putko P., Kwaśny M. (2020). Bioavailable silicon forms in dietary supplements. Bull. Mil. Univ. Technol..

[32] Farooq M.A., Ali S., Hameed A., Ishaque W., Mahmood K., Iqbal Z. (2013). Alleviation of cadmium toxicity by silicon is related to elevated photosynthesis, antioxidant enzymes; suppressed cadmium uptake and oxidative stress in cotton. Ecotoxicol. Environ. Saf..

[33] Song A., Li Z., Zhang J., Xue G., Fan F., Liang Y. (2009). Silicon-enhanced resistance to cadmium toxicity in Brassica chinensis L. is attributed to Si-suppressed cadmium uptake and transport and Si-enhanced antioxidant defense capacity. J. Hazard. Mater..

[34] Shi G., Cai Q., Liu C., Wu L. (2010). Silicon alleviates cadmium toxicity in peanut plants in relation to cadmium distribution and stimulation of antioxidative enzymes. Plant Growth Regul..

[35] Ping L., Xingxiang W., Zhang T., Dongmei Z., Yuanqiu H. (2008). Effects of several amendments on rice growth and uptake of copper and cadmium from a contaminated soil. J. Environ. Sci..

[36] Wang Y., Hu Y., Duan Y., Feng R., Gong H. (2016). Silicon reduces long-term cadmium toxicities in potted garlic plants. Acta Physiol. Plant..

[37] Shi X., Zhang C., Wang H., Zhang F. (2005). Effect of Si on the distribution of Cd in rice seedlings. Plant Soil.

[38] Mondal N.K., Chittaranjan D., Satinath Datta J.K., Arnab B. (2013). Effect of varying cadmium stress on chickpea (*Cicer arietinum* L) seedlings: An ultrastructural study. Ann. Environ. Sci..

[39] Imtiaz M., Tu S., Xie Z., Han D., Ashraf M., Rizwan M.S. (2015). Growth, V uptake, and antioxidant enzymes responses of chickpea (*Cicer arietinum* L.) genotypes under vanadium stress. Plant Soil.

[40] Guo L., Chen A., He N., Yang D., Liu M. (2017). Exogenous silicon alleviates cadmium toxicity in rice seedlings in relation to Cd distribution and ultrastructure changes. J. Soils Sediments.

[41] Khaliq A., Ali S., Hameed A., Farooq M.A., Farid M., Shakoor M.B., Mahmood K., Ishaque W., Rizwan M. (2016). Silicon alle-viates nickel toxicity in cotton seedlings through enhancing growth, photosynthesis, and suppressing Ni uptake and oxidative stress. Arch. Agron. Soil Sci..

[42] Li N., Feng A., Jiang Z., Wei S. (2020). Silicon application improved the yield and nutritional quality while reduced cadmium concentration in rice. Environ. Sci. Pollut. Res..

[43] Etesami H., Jeong B.R. (2018). Silicon (Si): Review and future prospects on the action mechanisms in alleviating biotic and abiotic stresses in plants. Ecotoxicol. Environ. Saf..

[44] Fujimaki S., Suzui N., Ishioka N.S., Kawachi N., Ito S., Chino M., Nakamura S.-I. (2010). Tracing Cadmium from Culture to Spikelet: Noninvasive Imaging and Quantitative Characterization of Absorption, Transport, and Accumulation of Cadmium in an Intact Rice Plant. Plant Physiol..

[45] Rehman M.Z.U., Rizwan M., Rauf A., Ayub M., Ali S., Qayyum M.F., Waris A.A., Naeem A., Sanaullah M. (2019). Split application of silicon in cadmium (Cd) spiked alkaline soil plays a vital role in decreasing Cd accumulation in rice (*Oryza sativa* L.) grains. Chemosphere.

[46] Keller C., Rizwan M., Davidian J.-C., Pokrovsky O.S., Bovet N., Chaurand P., Meunier J.-D. (2014). Effect of silicon on wheat seedlings (*Triticum turgidum* L.) grown in hydroponics and exposed to 0 to 30 µM Cu. Planta.

[47] Khan Z.S., Rizwan M., Hafeez M., Ali S., Adrees M., Qayyum M.F., Khalid S., Rehman M.Z.U., Sarwar M.A. (2020). Effects of silicon nanoparticles on growth and physiology of wheat in cadmium contaminated soil under different soil moisture levels. Environ. Sci. Pollut. Res..

[48] Liang Y., Sun W., Zhu Y.-G., Christie P. (2007). Mechanisms of silicon-mediated alleviation of abiotic stresses in higher plants: A review. Environ. Pollut..

[49] Ma J.F., Takahashi E. (2002). Soil, Fertilizer, and Plant Silicon Research in Japan.

[50] Qayyum M.F., Rehman M.Z.U., Ali S., Rizwan M., Naeem A., Maqsood M.A., Khalid H., Rinklebe J., Ok Y.S. (2017). Residual effects of monoammonium phosphate, gypsum and elemental sulfur on cadmium phytoavailability and translocation from soil to wheat in an effluent irrigated field. Chemosphere.

[51] Rios J.J., Ballesta M.M., Ruiz J.M., Blasco B., Carvajal M. (2017). Silicon-mediated Improvement in Plant Salinity Tolerance: The Role of Aquaporins. Front. Plant Sci..

[52] Abedi T., Mojiri A. (2020). Cadmium Uptake by Wheat (*Triticum aestivum* L.): An Overview. Plants.

[53] Abedi T., Mojiri A. (2020). Arsenic Uptake and Accumulation Mechanisms in Rice Species. Plants.

[54] Xu Q., Wang C., Li S., Li B., Li Q., Chen G., Chen W., Wang F. (2017). Cadmium adsorption, chelation and compartmentalization limit root-to-shoot translocation of cadmium in rice (*Oryza sativa* L.). Environ. Sci. Pollut. Res..

[55] Zhang C., Yin X., Gao K., Ge Y., Cheng W. (2013). Non-protein thiols and glutathione S-transferase alleviate Cd stress and reduce root-to-shoot translocation of Cd in rice. J. Plant Nutr. Soil Sci..

[56] Lukačová Z., Švubová R., Kohanová J., Lux A. (2013). Silicon mitigates the Cd toxicity in maize in relation to cadmium translocation, cell distribution, antioxidant enzymes stimulation and enhanced endodermal apoplasmic barrier development. Plant Growth Regul..

[57] Ma J., Cai H., He C., Zhang W., Wang L. (2015). A hemicellulose-bound form of silicon inhibits cadmium ion uptake in rice (*Oryza sativa*) cells. New Phytol..

[58] Liu C., Li F., Luo C., Liu X., Wang S., Liu T., Li X. (2009). Foliar application of two silica sols reduced cadmium accumulation in rice grains. J. Hazard. Mater..

[59] Nagata M., Yamashita I. (1992). Simple Method for Simultaneous Determination of Chlorophyll and Carotenoids in Tomato Fruit. Nippon Shokuhin Kogyo Gakkaishi..

[60] Jones J.B., Case V.W. (1990). Sampling, handling, and analyzing plant tissue samples. Soil Test Plant Anal..

[61] Chapman H. (1965). Cation-exchange capacity. Methods of Soil Analysis: Part 2 Chem. Microbiol. Prop..

[62] Ma J.F., Tamai K., Ichii M., Wu G.F. (2002). A Rice Mutant Defective in Si Uptake. Plant Physiol..

[63] Mahmood A., Malik R.N. (2014). Human health risk assessment of heavy metals via consumption of contaminated vegetables collected from different irrigation sources in Lahore, Pakistan. Arabian J. Chem..

[64] Hamid A., Wasim A., Azfar A., Amjad R., Nazir R. (2020). Monitoring and health risk assessment of selected trace metals in wheat rice and soil samples. Food Sci. Technol..

[65] United States Environmental Protection Agency (2000). Risk-Based Concentration Table.

